# Linking e-health records, patient-reported symptoms and environmental exposure data to characterise and model COPD exacerbations: protocol for the COPE study

**DOI:** 10.1136/bmjopen-2016-011330

**Published:** 2016-07-13

**Authors:** Elizabeth Moore, Lia Chatzidiakou, Roderic L Jones, Liam Smeeth, Sean Beevers, Frank J Kelly, Jennifer K Quint, Benjamin Barratt

**Affiliations:** 1 Department of Medicine, Imperial College London, London, UK; 2 Department of Chemistry, Centre for Atmospheric Science, University of Cambridge, Cambridge, UK; 3 Department of Epidemiology and Population Health, London School of Hygiene & Tropical Medicine, London, UK; 4 Analytical & Environmental Sciences Division, King's College London, London, UK; 5 NIHR Health Protection Research Unit in Health Impacts of Environmental Hazards, King's College London, London, UK

**Keywords:** COPD, Exacerbation, Pollution, Monitor

## Abstract

**Introduction:**

Relationships between exacerbations of chronic obstructive pulmonary disease (COPD) and environmental factors such as temperature, humidity and air pollution are not well characterised, due in part to oversimplification in the assignment of exposure estimates to individuals and populations. New developments in miniature environmental sensors mean that patients can now carry a personal air quality monitor for long periods of time as they go about their daily lives. This creates the potential for capturing a direct link between individual activities, environmental exposures and the health of patients with COPD. Direct associations then have the potential to be scaled up to population levels and tested using advanced human exposure models linked to electronic health records.

**Methods and analysis:**

This study has 5 stages: (1) development and deployment of personal air monitors; (2) recruitment and monitoring of a cohort of 160 patients with COPD for up to 6 months with recruitment of participants through the Clinical Practice Research Datalink (CPRD); (3) statistical associations between personal exposure with COPD-related health outcomes; (4) validation of a time-activity exposure model and (5) development of a COPD prediction model for London.

**Ethics and dissemination:**

The Research Ethics Committee for Camden and Islington has provided ethical approval for the conduct of the study. Approval has also been granted by National Health Service (NHS) Research and Development and the Independent Scientific Advisory Committee. The results of the study will be disseminated through appropriate conference presentations and peer-reviewed journals.

**Strengths and limitations of this study**

- This study will allow researchers to assess associations in far more detail, initially at the individual patient level and potentially at a national level.
- It will demonstrate the integration of novel methodological approaches in three main areas: (1) the recruitment of participants via an anonymised general practice records database, and use of electronic health records to gather information on chronic obstructive pulmonary disease (COPD) exacerbations; (2) mass deployment of portable air quality sensor platforms over long periods revolutionising the way in which personal exposure can be quantified and (3) the application of a dynamic human exposure model.
- Much of the success depends on participant participation over a long period (up to 6 months) and there may be difficulties with recruiting enough participants to power the study.
- Physiological and inflammatory changes are not being recorded as part of this study; however, these issues will be addressed in this protocol and will be examined in a substudy of characterisation of COPD exacerbations using environmental exposure modelling.

## Introduction

Chronic obstructive pulmonary disease (COPD) is a chronic progressive disease associated with the abnormal inflammatory response of the lungs to noxious particles or gases^1^ and is characterised by increased resistance to airflow in small conducting airways, changes in lung compliance and the premature collapse of airways during expiration.^2^ The inflammatory responses can lead to increased sputum production, breathlessness and reduced lung function, often resulting in reduced exercise tolerance and decreased quality of life.^3^ ^4^ COPD has a large burden on healthcare resources with an estimated annual cost to the National Health Service (NHS) currently of over £800 million.^5^ At present, it is the fourth leading cause of death worldwide, and it is predicted that total deaths from COPD may increase by more than 30% in the next 10 years unless urgent action is taken to reduce the underlying risk factors.^6^

Smoking is the most important risk factor for COPD; however, an estimated 25–45% of patients have never smoked. Other risk factors include a history of pulmonary tuberculosis, chronic asthma, childhood respiratory tract infections, occupational exposure to dusts and gases, air pollution and low socioeconomic status.^7^ The prevalence of COPD is increased in individuals living close to traffic,^8^ and patients with COPD have substantial mortality risks associated with particles^9^ and temperature changes.^10–12^ Exacerbations of COPD are acute episodes of deterioration associated with increased mortality and decreased quality of life, and are the second most common cause of adult emergency medical hospital admission in the UK.^8^ Infections, both bacterial and viral, are known to play a major role in exacerbations.^4^

Gaps still exist in our understanding of the mechanisms involved in exacerbations and the particular air pollutants and environmental conditions that lead to increased hospitalisations. Previous systematic reviews and meta-analytic studies have found small but significant effects of particulate matter (PM_10_ and PM_2.5_) and gases such as ozone (O_3_) and nitrogen dioxide (NO_2_) on COPD-related admissions and mortality.^13–17^ However, such findings are only indicative, as the evidence comes from a relatively small number of time-series and case-crossover studies with significant heterogeneity between them. The methodological design of those studies introduced additional limitations in the interpretability of the findings stemming from the inability to accurately characterise exposure to air pollutants at the individual level. Such critical limitations have been the absence in most studies of detailed activity patterns, the reliance on aggregated health counts and the low spatiotemporal resolution of air pollution from a small number of fixed monitoring sites resulting in the inadequate adjustment for confounders and covariance between air pollutants.

Consequently, there has been a continued effort to understand the relationship between ambient concentrations and personal exposure. Personal exposure assessment requires the recording of a person's time-activity patterns, as well as the pollutant concentrations which each individual is exposed to. At the most basic level, this may be the relative proportion of time spent in different microenvironments. Additionally, activity type of individuals may affect indoor air pollution levels, while activity levels may alter dose. Estimating personal exposure has been challenging, because of the expense and availability of personal monitors, as well as the lack of detailed information at the individual level which is limited by the accuracy of time-activity diaries, which can be laborious, introduce recall biases and reliability, and require active cooperation of the participants in the monitoring process, often limiting their application in small panel studies.

This research is timely as it brings together recent advancements in technological aspects of personal air quality monitors and computational developments to create detailed hybrid models of personal exposure. This paper presents the integrated methodological framework which will be used for the ‘characterisation of COPD exacerbations using environmental exposure modelling’ (COPE) study. This research project takes the first steps towards the integration of novel methodological approaches in three main areas : (1) the recruitment of participants via an anonymised general practice records database, and use of primary care electronic records to gather information on COPD exacerbations; (2) mass deployment of portable air quality sensor platforms over long periods revolutionising the way in which personal exposure can be quantified with automated classification of individual time-activity patterns and exposure events and (3) the application of a dynamic human exposure model. Together, these have the potential to provide powerful tools to create and validate accurate personal exposure models with higher spatiotemporal resolution, allowing, for the first time, the incorporation of spatially realistic exposure models in epidemiological studies.

## Methods and analysis

**Figure 1:**
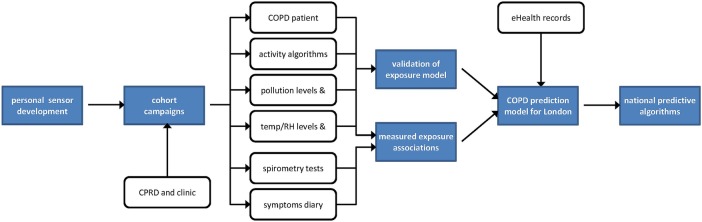
Project flow diagram. COPD, chronic obstructive pulmonary disease; CPRD, Clinical Practice Research Datalink; RH, relative humidity.

A series of five work packages move through a number of phases, from instrument development and recruitment, through cohort monitoring and analysis, to predictive model development (figure 1).

### Development and long-term deployment of personal air pollution sensors

**Figure 2:**
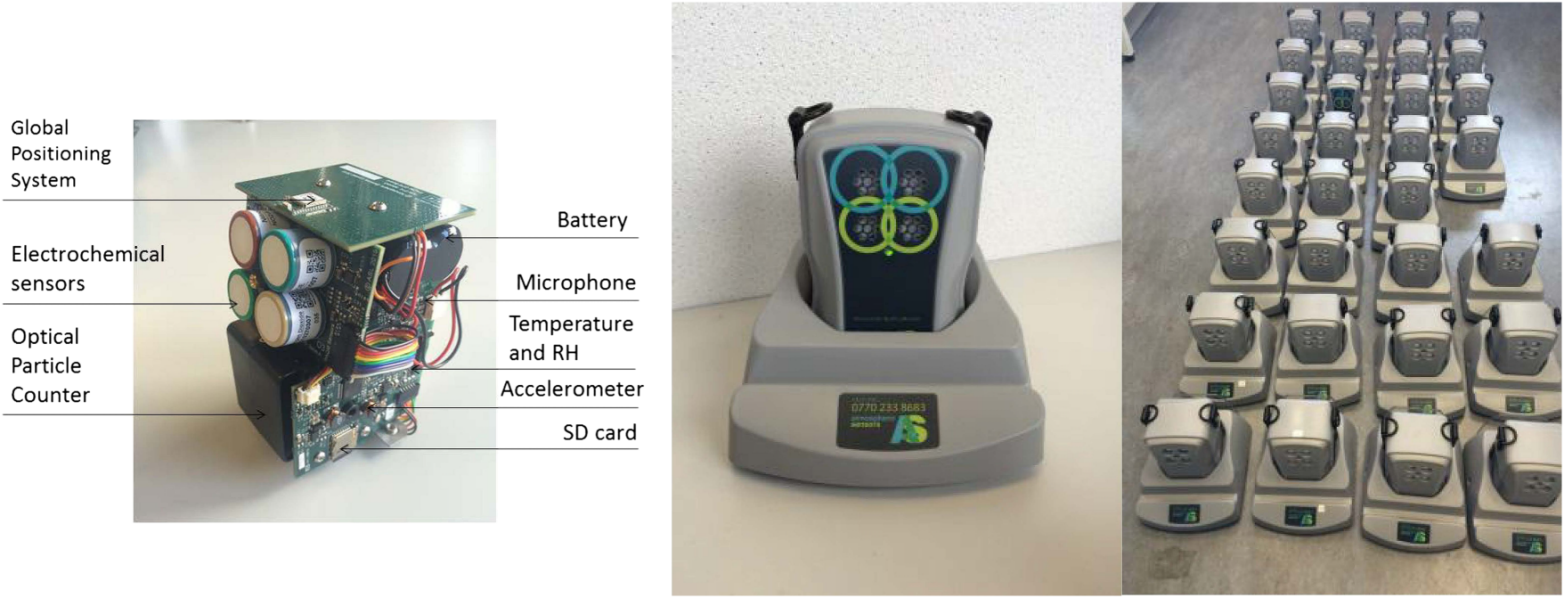
Design of the PAM platform internals, in charging base-station and ‘en masse’. PAM, personal air monitor; RH, relative humidity; SD, secure digital.

Personal air monitors (PAMs) have been designed, manufactured and tested specifically for the COPE study (figure 2). The PAMs can be either strapped around the waist with a belt or worn over the shoulder. A waterproof case will be provided to the participants to make it less conspicuous when worn outside the house. The PAMs will employ ubiquitous sensing of a large number of geotemporal environmental parameters that can be measured simultaneously (table 1). The measurements will be stored in the sensor and uploaded through General Packet Radio Service to a secure server through the charging base station. No interaction with the unit is required by the participant, other than to place it in its charger each night (the battery life of the sensor is 30 hours between charges). It will operate continuously and is almost silent.

**Table 1:** Summary of monitored parameters of the PAMs

Parameter	Method	Monitoring interval
Spatial coordinates	GPS	20 s
Background noise	Microphone	100 Hz
Physical activity	Triaxial accelerometer	100 Hz
Temperature (°C)	Thermocouple	20 s
RH (%)	Electrical resistive sensor	20 s
PM_1_, PM_2.5_, PM_10_ (μg/m^3^)	OPC	20 s
CO, NO, NO_2_, O_3_ (ppb)	Electrochemical sensors	20 s

CO, carbon monoxide; GPS, Global Positioning System; NO, nitric oxide; NO_2_, nitrogen dioxide; OPC, optical particle counter; O_3_, ozone; PAM, personal air monitor; PM, particulate matter; ppb, parts-per-billion; RH, relative humidity.

In order to reduce transmission costs and the computational burden of the portable device, transmitted data from the accelerometer and microphone will be reduced by event counting within 20 s non-overlapping windows. Spatial points resulting from Global Positioning System (GPS) coordinate errors were identified and have been removed based on Euclidean distance and earth bearing between consecutive points.

The selected gases (NO_2_, O_3_, NO and CO) will be quantified with electrochemical sensors based on amperometric methods at parts-per-billion (ppb) mixing ratios. Once appropriate calibration factors and postprocessing have been applied to sensor data, excellent sensitivity can be achieved in laboratory and field settings.^18^ The PAM incorporates a miniaturised optical particle counter that will record particle counts in 16 particle sizes (bins) in the range from 0.35 to >17 μm. The bins will then be aggregated to estimate the mass of the three fractions PM_1_, PM_2.5_ and PM_10_.

### Participant recruitment and monitoring

#### Recruitment of panel participants

Traditionally, recruitment for observational studies involves time-consuming and labour-intensive contact with suitable participants that meet the inclusion/exclusion criteria. In this study, we employ a novel method of recruitment that involves approaching GPs and patients to participate through the Clinical Practice Research Datalink (CPRD), an anonymised general practice records database containing ongoing primary care medical data. This method of recruiting for observational and interventional studies has been shown to be effective in a pharmacogenetic study;^19^ and in a cluster randomised control trial on asthma exacerbation among school-aged children.^20^ Apart from the efficiency in recruiting participants, this method can also be considered broadly representative of the UK general populations with coverage of over 11.3 million patients and 674 practices.^20^ An additional benefit is that once participants are recruited, the anonymous data from electronic health records (EHRs) can be linked to diverse parameters collected simultaneously (eg, data from air quality monitors/mobility data) to provide detailed clinical information about the study participants.

In total, 160 participants will be recruited from CPRD using an algorithm containing validated COPD diagnostic codes. Patients with data in CPRD who have a diagnosis of COPD based on a validated code list by Quint *et al*^21^ are not coded for mild COPD (ie, moderate or severe patients only), are not coded as a current smoker, are aged >35 years, and have had between one and two identified exacerbations in the preceding year will be included. After running the algorithm to identify suitable participants, general practices that have agreed to participate in research through CPRD will be sent a list of the potential participants. GPs will confirm to CPRD the suitable patients identified previously using the Vision Identification. CPRD will then send participant information packs to the general practices to disseminate to the potential recruits. The information pack will contain a cover letter from the general practitioner introducing the study, a participant information sheet with detailed information of what the project entails, and an expression of interest form that participants can complete and send to the research team in a prepaid envelope. Once received, the research team will then be able to contact the participant to enrol them in the study through a clinic appointment. Participants will also be recruited from respiratory clinics in secondary care as an additional recruitment option.

The sample size of 160 patients is based on the estimated number of exacerbations for the cohort. Since we will recruit with a bias towards patients with COPD with a history of COPD exacerbations, we have made the conservative estimate that we will capture at least 200 exacerbations with a cohort of 160 patients. We have calculated a minimum detectable relative risk (RR) to detect associations at p<0.05 with 80% power. With 200 exacerbations, this will permit detection of about RR=1.65 in the highest 20% of days compared with others (RR=2.00 in the highest 10%). Other more common outcomes and in particular peak flow will have power to detect smaller associations.^22^

Two secondary recruitment methods will be established to make up any shortfall in recruits through the CPRD: recruitment from respiratory clinics in local hospitals and presentations at British Lung Foundation ‘Breathe Easy’ respiratory disease support groups.

#### Monitoring phase

At the clinic, participants will be provided with a PAM and instructed to keep the monitor at home and take it out with them for a minimum of once a week for up to 6 months. An initial questionnaire will collect information on lifestyle factors and residence characteristics, such as type of cooker used in the home (eg, gas, electric or wood burning stove) and car ownership. During the study period, participants will complete daily diary cards of their symptoms, any changes to their treatment (eg, medications) and sleep disturbance. They will be asked to record their peak expiratory flow on the diary card using a peak flow meter. Spirometry readings will be collected at the initial appointment and subsequent follow-up visits if the participant consents. This will provide information on the severity of their condition and may control for possible random differences.

If at any stage the wearing compliance of the PAM is low, or the participant chooses to withdraw, a replacement will be recruited. Throughout the monitoring period, participants will receive phone calls from the research assistant to check how they are coping with the study. Six weeks into the monitoring period, participants will be invited to attend a clinic with the research assistant to discuss any issues with the PAMs or diary cards. At the end of the monitoring period (at 6 months or earlier if the participant wishes to withdraw), participants will be invited to a final appointment to return the PAMs and completed diary cards.

#### Use of anonymised EHR from CPRD

This is a consented study and, as such, participants will be asked to give their consent in the first appointment to the use of their anonymised data in the research analysis involved. CPRD will provide GOLD data sets to the chief investigator at Imperial College London which are then downloaded from the clinical IT system. Data will be stored against a ‘non-identifying’ identifier (first level anonymised) generated using a key held only by the general practice, so that they cannot be linked back to the data sets using Imperial College London's online access. A second key will be used to generate a further level of anonymisation at the CPRD data centre before any data are seen by researchers undertaking any aspect of the trial analysis. In order to comply with CPRD's approval from the Confidentiality Advisory Group of the Health Research Authority, there will be a reidentification risk management plan in place to prevent de-anonymising the CPRD. Since there is a way back via the two keys to check the validity of the data, this is technically a pseudoanonymisation. At the research end, however, patient data are effectively fully anonymised as there is no way that a researcher can obtain access to either of the two keys which are held securely in two different locations. The research assistant will only have access to the raw patient data (collected from the daily symptom diary cards, spirometry readings and questionnaire) and those performing the analysis will only have access to anonymised data. All data at whatever location will be stored in systems that fully meet all data storage requirements and have appropriate standard operating procedures.

### Statistical associations between personal exposure with COPD-related health outcomes

The monitoring phase of the project will create a unique high resolution multiparameter data set of individual exposure patterns over an extended period. These data will be mined to explore associations between participant's health (symptoms, lung function and exacerbations) and the environment through (1) direct measurements, (2) derived variables and (3) modelled outputs. Explanatory variables will include peak, mean and cumulative exposure, rate of change, activity level and pollutant dose/intake, lag effects, ambient pollution or temperature episode effects, pollutant source types (eg, traffic, regional, domestic) and indoor/outdoor ratios. The aim will be to identify and explain any observed associations, allowing the translation of results into healthcare relevant information and possible policy updates.

### Statistical analysis

Survival analyses for repeated measurements will be performed on the basis of the Cox proportional hazards model. Interval censoring for handing ties over appropriate event time intervals will be applied specifying each participant to a stratum. Essentially, conditional regression will be used to estimate the HR of subjective health symptoms and exacerbations in relation to seasonal variations of personal exposure (prognostic factor). The conditional regression models will be developed by the PHREG procedure in SAS V.9.3 (North Carolina, USA) with robust sandwich covariance for aggregated data. Ties will be handled with the DISCRETE method. Medication use will be inserted as a control factor in the models.

### Use of EHR to analyse exacerbations

**Figure 3:**
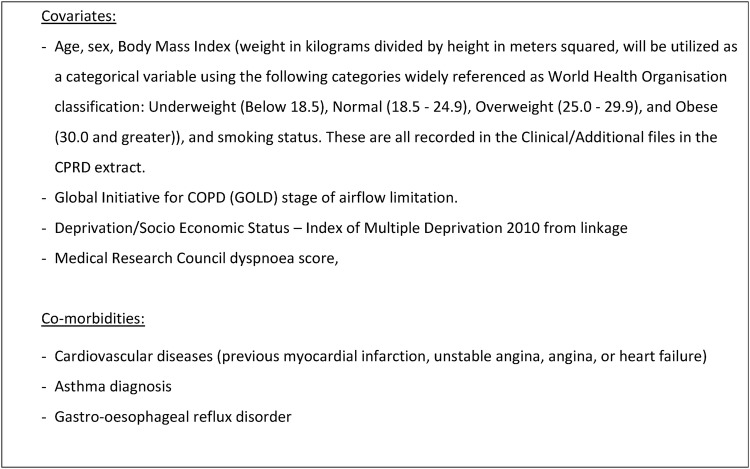
Covariates and comorbidities to be obtained from EHR. COPD, chronic obstructive pulmonary disease; CPRD, Clinical Practice Research Datalink; EHR, electronic health record.

CPRD GOLD data sets will be used to identify general practitioner-treated COPD exacerbations. Information from Hospital Episode Statistics (HES) and the Office of National Statistics (ONS) will also be gathered from CPRD including: accident and emergency admissions, hospital admissions and mortality. Mortality data from ONS will be used as a severity index for exacerbation. Figure 3 shows the covariates and comorbidities to be used in the statistical analysis of COPD exacerbations. Spatial and temporal patterns of recorded exacerbations extracted from historical CPRD, HES and ONS records will be compared with model risk estimates, with the aim of deriving predictive algorithms for future hospitalisations.

### Activity algorithms

Time-location-activity patterns of individuals are an important determinant of personal exposure to air pollution. In this study, we will derive activity pattern combining personal sensing technology with machine learning computational techniques for automated classification and without recourse to manual activity diaries. This method is currently being validated by the University of Cambridge in a pilot cohort of 45 healthy volunteers over a week as they go about their daily lives, each of whom will keep a detailed smartphone-based activity diary. The automated classification of exposure events will provide improved estimation of personal exposure and dose which will be used to draw associations with subjective symptoms (diary cards), measured outcomes (peak flow and general practice/hospital records from CPRD) and medication use.

### Validation of the London Hybrid Exposure Model

King's College London has previously developed a time-activity exposure model study known as the London Hybrid Exposure Model (LHEM),^23^ but full evaluation against measured data was not possible at the time due to limitations in mobile monitoring technology. The extensive measurement data set gathered will primarily provide a validation data set for the LHEM. The GPS coordinates collected with the PAMs, together with the automated classifications of time-activity models created, will be fed into the model. This will produce a modelled time-series exposure estimate for each pollutant. These estimates will be compared with measured pollutant exposure and performance for targeted pollutants in different microenvironments, with the aim of deriving uncertainty estimates for future model applications. Calibrated model results will be compared with static exposure estimate methodologies, such as central monitor or postcode, to quantify the exposure misclassification associated with each. The integrated data set collected during the monitoring phase will also provide the opportunity to verify and refine model infiltration factors for indoor and transport microenvironments, incorporating emissions from indoor sources and human activities, such as cooking and smoking.

This combined monitoring–modelling methodology for time-activity exposure model development and evaluation will be applicable to a wide range of cohort and epidemiological studies investigating links between environmental exposure and diverse health outcomes.

### Development of a COPD prediction model

Associations between exacerbations with spatiotemporally resolved environmental exposure established in the Statistical associations between personal exposure with COPD-related health outcomes section will be combined with the time-activity exposure model (LHEM) to create a predictive model for COPD exacerbations across London. First, time-location-activity patterns of the COPD cohort will be compared with general population-level time-activity patterns derived from the Traffic Pollution and Health in London project.^24^ This will be used to test the applicability of general population behaviour patterns when assessing COPD associations in epidemiological studies.

The association between personal exposure to air pollution with COPD exacerbations estimated in the Statistical associations between personal exposure with COPD-related health outcomes section will form the basis of the COPD prediction model. This model will be used to create high resolution (20×20 m grid) daily COPD exacerbation risk maps for the years 2005–2011, based on modelled meteorological and pollutant conditions, coupled with typical patient with COPD time-activity patterns identified in the Activity algorithms section. The model will thus retrospectively predict days and locations more likely to trigger a worsening of symptoms and/or exacerbation in patients with COPD over this time period.

The performance of the predictive model will be evaluated using validated methods for patient identification^21^ from EHRs, CPRD, HES and ONS death data excluding data from the COPE cohort. Spatial and temporal patterns of recorded exacerbations will be compared with model risk estimates linked to the home address. If it is demonstrated that there are significant associations between model predictions and recorded exacerbations, the algorithms used will be generalised for use outside of London. These algorithms will provide an opportunity for the development of a national COPD forecasting service with proven performance in predicting increased risk of exacerbations.

## Discussion

Several studies have attempted to identify relationships between environmental factors and COPD exacerbations.^25^ ^26^ However, limitations of the methodological design of previous studies have made it difficult to identify clear links between exposure and health outcomes.

The strength of this study lies in the fact that we will have the ability to assess these associations in far more detail, initially at the individual patient level and potentially at a national level.

For the first time, this study will provide a multidisciplinary methodological framework that will bring together recent advancements in low-cost wearable sensors, computational techniques for the estimation of activity-weighted personal exposure and advanced spatial mapping in a well-characterised population study. The integrated database of environmental stressors and activity patterns at the individual level will form the basis for the validation of the LHEM. The LHEM can further incorporate spatial and temporal patterns of recorded exacerbations extracted from historical CPRD, HES and ONS records to form a COPD prediction model for future hospitalisations.

Limitations include the fact that participants will be asked to participate in the study for up to 6 months in order to try and capture seasonal changes in exacerbations and some may be dissuaded by this. Despite the process of identifying and recruiting patients via CPRD, there may be insufficient numbers of patients who are interested in participating to power the study. Owing to these recruitment concerns, we are not planning to request blood and sputum samples from all participants, and this means that we will not have any biological data from some participants to assess physiological and inflammatory changes during their exacerbations. However, funding has been obtained for a substudy within COPE to collect blood and sputum samples in a subset of 20 participants at baseline, exacerbation and 4 weeks post exacerbation.

The methodology presented here will allow development of forecasting models that can be used to predict times of increased exacerbation risk. This will aid healthcare providers and allow more accurate planning and allocation of resources which will reduce costs for the NHS. It will aid patients as it may provide an opportunity to alter behaviour and to prevent exacerbations from occurring. By providing a more robust evidence base, policymakers may be able to take more targeted and efficient decisions on reducing environmental risk. Members of the public will be able to make more informed decisions on how to minimise their own risks, improving health and quality of life.

## Ethics and dissemination

CPRD has been granted Multiple Research Ethics Committee (MREC) approval to undertake observational studies and external data linkages with HES and ONS. Research and Development (R&D) approval has also been granted by the Royal Brompton and Harefield NHS Foundation Trust and Guy's and St Thomas' NHS Foundation Trust to carry out the study at the Clinical Research Facility in the Royal Brompton Hospital and the Lane Fox Respiratory Unit at St Thomas' Hospital (IRAS ref 166785). Ethical approval is being sought to collect blood and sputum samples from a subset of 20 participants for a pilot study.

Participants will be informed that the monitors use GPS technology and will provide spatial data at intervals during the day. Participants will be reassured that this information will only be accessed at the end of the study to analyse overall spatial and temporal relationships and not real-time movement. In addition, participants will be informed that the monitors will have a built-in microphone for the purpose of recording ambient (background) noise levels. It will not be used for the recording of speech.

The behavioural and environmental COPD associations identified during the study will be adapted for application on a national scale and disseminated to healthcare providers including the Department of Health, Clinical Commissioning Groups and GPs. Information will include evidence of environmental conditions and patient activities that are found to contribute to an increased risk of COPD symptoms and exacerbations. The predictive algorithms will be made available, allowing the development of a validated national COPD forecasting system. Such a system has the potential for further commercial exploitation and the research team will seek to collaborate with the UK Meteorological Office in order to improve the effectiveness of their Healthy Outlook service. This research would provide an opportunity to carry out a cost–benefit analysis of such a system, now essential for commissioning in the current health market.
